# Beneficial effects of Alterion supplementation on growth metrics, intestinal histomorphology, and microbial communities in indigenous and commercial chicken breeds

**DOI:** 10.3389/fvets.2025.1630712

**Published:** 2025-07-11

**Authors:** Waleed Al-Marzooqi, Ahmed Elaswad, Hani M. El-Zaiat, Yasmin ElTahir, Kaadhia Al-Kharousi, Syed K. Hassan

**Affiliations:** ^1^Department of Animal and Veterinary Sciences, College of Agricultural and Marine Sciences, Sultan Qaboos University, Muscat, Oman; ^2^Center of Excellence in Marine Biotechnology, Sultan Qaboos University, Muscat, Oman; ^3^Department of Animal Wealth Development, Faculty of Veterinary Medicine, Suez Canal University, Ismailia, Egypt; ^4^Product Development Department, Oman Flour Mills Company (S.A.O.G), Ruwi, Oman

**Keywords:** Alterion, chickens, growth performance, gut microbiome, intestinal morphology

## Abstract

This study evaluated the effects of Alterion supplementation on growth rate, feed conversion ratio, intestine morphology, carcass quality, and blood indices in both commercial and local chicken breeds. Two chicken breeds (Local Omani and Cobb 430 broilers) and two dietary treatments (Control and 0.05% Alterion) were used in a 2 × 2 factorial design. The results showed that, across both breeds, supplementation significantly improved weight gain over 42 days compared to the control. Specifically, the Alterion group exhibited a 12.1% increase in Cobb 430 and a 26.7% increase in Omani birds, with all differences being statistically significant (*p* < 0.001). Furthermore, the jejunum and ileum of both breeds fed supplemented diets exhibited higher villus height and villus-to-crypt ratio than the control group (*p* < 0.05). The counts of red blood cells (RBCs), white blood cells (WBCs), and total protein increased significantly in both chicken breeds fed supplemented diets compared with controls (*p* < 0.05). Carcass and internal organs were remarkably larger in Cobb 430 than in Omani chickens (*p* < 0.05), and in Alterion treatments than in controls (*p* < 0.05). In both chicken breeds, meat quality parameters were not significantly affected by Alterion. Alterion supplementation modulated gut microflora composition and relative abundance, with Bacilli being the most abundant class in all treatments and gut segments (*p* < 0.05). While Alterion supplementation had minimal influence on the overall composition of the bacterial community, it contributed to maintaining a normal ecological balance of the microbiota. In summary, supplementation with 0.05% Alterion improved growth, intestinal health, blood parameters, carcass yield, and internal organ weight, and beneficially modulated the gut microbiome in Cobb 430 and Omani chickens. Further research is recommended to determine the optimal dosage of Alterion for Omani chickens, thereby optimizing their performance.

## 1 Introduction

Microbial colonization of the chicken gastrointestinal (GI) tract begins shortly after hatch (1). The establishment of intestinal microbiota typically starts within the first 2 to 4 days post-hatching, with the microbial community in the small intestine becoming relatively stable by approximately 2 weeks of age. Conversely, the cecal microbiota may take up to 30 days to fully mature (2). The composition of these microbial populations is not static; rather, it undergoes continuous changes influenced by multiple factors such as the bird’s age and nutritional intake (3). Significant differences in microbial communities occur across various regions of the GI tract (4), with each section hosting a specific microbiota profile. Diversity and complexity increase with age, feed composition, genetic background, and environmental origin (2, 5–7). In recent years, there has been heightened research interest in microbial development across different chicken genotypes (8). Despite this, knowledge remains limited regarding the variability of gut microbiota among genetically distinct chicken lines, particularly in slow-growing types like indigenous breeds.

The gastrointestinal microbiota is a key contributor to host metabolic processes, nutrient assimilation, growth efficiency, and overall health status (9). Maintaining optimal gut health in poultry production is crucial for achieving maximum performance and ensuring bird welfare, as microbial imbalances may trigger inflammatory responses and gastrointestinal disorders (10). Several studies have documented the use of probiotics as a strategy to stabilize or restore the intestinal microbiota (11, 12). The proposed health benefits of probiotics include mitigating disease risk, potentially limiting the colonization and proliferation of pathogenic microorganisms, preserving microbial homeostasis, and enhancing immune defense mechanisms (13–15). In the poultry industry, probiotic supplementation has also been associated with improved growth metrics and favorable carcass characteristics (16). However, to date, there appears to be a lack of comprehensive investigations examining the impact of probiotics on production traits and gut microbial diversity in indigenous Omani chicken breeds. While extensive research exists on the genetic determinants of growth performance in commercial broiler strains, these findings may not apply directly to slower-growing native chicken populations.

Monitoring the composition of intestinal microflora is of significant importance, as various bacterial species with pathogenic potential for humans have been identified within the GI tract of chickens, posing a risk of transmission through the food chain (17, 18). Gaining insight into the development of a stable and healthy gut microflora enables the identification of disturbances within the microbial community and facilitates the assessment of how changes in poultry management practices influence gut ecology. Such knowledge offers the potential to deliberately alter the gut microbiota to enhance gut health and optimize feed conversion ratio (FCR) (9, 19). Several factors are known to influence the structure and function of avian gut microbial communities, including diet composition (20), age (5), and environmental conditions (21). Accordingly, this study explored the effect of probiotic supplementation on growth performance, feed consumption, FCR, hematological and serum biochemical profiles, meat quality, intestinal histomorphology, and gut microbial composition in indigenous and commercial chicken strains.

## 2 Materials and methods

### 2.1 Experimental design

The 2 × 2 factorial designs included two chicken breeds (Local Omani and Cobb 430-type broilers) and two dietary treatments: basal diet and Alterion (basal diet containing 0.05% Alterion® *Bacillus subtilis* 29,784), as recommended by the manufacturer (Adisseo, France), resulting in four experimental treatments: Control Omani, Control Cobb, Alterion Omani, and Alterion Cobb.

### 2.2 Birds and housing

Upon arrival, all chicks were individually weighed and sorted into specific weight ranges to ensure uniformity; individuals exhibiting extreme body weights (either too low or too high) were excluded from the experiment. A total of 440-day-old chicks from each breed were utilized in this study. Chicks were randomly allocated in groups of five to each of 88 suspended wire cages, ensuring that the initial average body weight per cage was approximately equal. Feed and water were made available *ad libitum* throughout the experimental period. The cages were housed indoors in an environment-controlled facility, where the temperature was maintained at 33°C on day 1, with a weekly drop of 3°C until it reached 22°C. A lighting regimen of 23 h of light and 1 h of darkness was applied.

### 2.3 Experimental diets and feeding

Birds were offered their designated experimental diets, formulated and administered in mash form. The feeding regimen comprised a starter phase (the first 21 days) and a finisher phase (from day 22 to day 42). All feed formulations (Table 1) were produced without antibiotics or feed additives at Atyab Food Tech Company (Oman). Following initial grouping, chicks from each breed were randomly assigned to one of two treatments: either a control group or a treatment group. Each group comprised 22 replicate cages, with five birds allocated to each cage. The assignment of treatment-replicate combinations was randomized. Body weight, feed intake, and feed conversion ratio (FCR) were measured every week. All birds and remaining feed in each cage were weighed on days 0, 7, 14, 21, 28, 35, and 42.

**Table 1:** Composition and chemical analysis of the basal diet.

Ingredients*	Starter	Finisher
Maize	54.138	61.139
Soybean meal	38.53	29.38
Canola oil	1.84	2.01
Monocalcium phosphate	0.84	0.65
Limestone	1.39	1.37
Salt	0.20	0.18
Pellet binder	1.00	3.00
DL-methionine	0.32	0.34
Choline mix	0.60	0.60
Sodium sulfate	0.20	0.23
Lysine HCl	0.126	0.278
L Threonine	0.126	0.143
Premix	0.69	0.68
Chemical analysis %		
Crude protein	22.80	19.40
Metabolizable energy (kcal/kg)	2,980	3,100
Ether extract	4.01	4.35
Crude fiber	3.01	3.35
Calcium	0.95	0.90
Phosphorus	0.45	0.40

*Each kg of diet was supplemented with 12,500 I. U of vitamin A, 3000 I. U of vitamin D3, 50 mg of vitamin E, 2 mg of vitamin K3, 2 mg of thiamin, 4 mg of riboflavin, 1 mg of pyridoxine, 0.01 mg of cyanocobalamin, 15 mg of pantothenic acid, 50 mg of folic acid, 10 mg of biotin, 450 mg of choline chloride, 1 mg of iodine, 0.3 mg of selenium, 40 mg of iron, 120 mg of manganese, 100 mg of zinc, and 1.5 mg of copper.

### 2.4 Carcass and visceral organ weights

One bird per cage was selected randomly from each breed/dietary treatment and euthanized on day 42 as described below (Section 2.7). The body weight before slaughter, as well as the weights of the carcass and visceral organs, were measured.

### 2.5 Assessment of meat quality

One bird per cage was randomly selected from each breed/dietary treatment to evaluate meat quality characteristics. The *M. pectoralis* major muscle was excised carefully from the breast region of each carcass. Cooking loss, muscle pH, (WB) Warner Bratzler-shear force, and color values a* (redness), b* (yellowness), and L* (lightness) were analyzed as previously described by Al-Marzooqi et al. (22).

### 2.6 Blood and biochemical markers

Following the procedure reported by Al-Aufi et al. (23), one bird per cage from each breed/dietary treatment was randomly selected for blood sample collection on day 42. Blood indices and biochemical parameters were analyzed according to the previously reported methodological guidelines (23).

### 2.7 Bird sedation and euthanasia for sample collections

A combination of 10% ketamine and 20% xylazine was administered via intramuscular injection to induce a state of deep sedation and anesthesia in the selected birds before procedures at different age periods. Once complete immobilization was achieved, euthanasia was carried out through cervical dislocation (22).

#### 2.7.1 Analysis of gut morphology

On day 42, the jejunum and ileum from two birds per cage from each breed/dietary treatment were examined histologically as described previously (22). A 3 cm portion was collected from the central region of each intestinal segment and preserved in 10% neutral-buffered formalin for subsequent analysis. Following fixation, the samples were rinsed with phosphate-buffered saline (PBS), embedded in paraffin, and sectioned at a thickness of 5 μm. The histological preparation involved two stages: embedding in low-melting-point paraffin wax and staining with hematoxylin and eosin. Morphometrics of intestinal morphology were analyzed using Image-Pro Plus 6.0 software (Media Cybernetics Inc., Bethesda, MD). For each intestinal sample, six independent measurements were taken for each parameter and averaged. Villus height (VH) was determined by measuring the distance between the villus’ apex and the lamina propria, whereas crypt depth (CD) was defined as the distance between the crypt base and the villus-crypt interface. All morphological measurements were conducted at 10 μm intervals using Image-PRO® PLUS 6.0 (Media Cybernetics Inc., Bethesda, MD).

#### 2.7.2 Metagenomics study

On days 21 and 42, luminal contents from the jejunum and ileum of nine birds per breed per treatment, per intestinal segment, and per timepoint (totaling 78 samples per breed) were collected for DNA extraction using the QIAamp DNA Stool Mini Kit (QIAGEN, Hamburg, Germany). The V3–V4 hypervariable segments of the 16S rRNA gene were amplified using universal primers as described by Al-Marzooqi (24). PCR reaction composition, thermal cycling, and amplicon purification were performed following the protocol by Al-Marzooqi (24). The purified amplicons were sent to BGI Genomic Laboratory (China) for high-throughput sequencing using the Illumina MiSeq platform.

### 2.8 Bioinformatics analysis

The reads were inspected, filtered, and trimmed to retain those with a Phred quality score of 20 or higher. The qualified reads were assembled to form tags for clustering into operational taxonomic units (OTUs). QIIME (version 1.8) was used to explore the OTUs. Using the SILVA database (SILVA version 1.8), the defined OTUs were grouped into different taxonomic levels. The alpha-diversity indices were computed using MOTHUR (v.1.31.2) and QIIME (v1.8.0). The alpha-diversity indices calculated in this study include several key measures of community diversity and richness, such as the Chao and ACE richness estimators, the Shannon index, the inverse Simpson diversity index, and the Observed Species. Moreover, Good’s coverage estimator was used to assess the proportion of diversity captured by the sequencing process and estimate sequencing depth. Bray–Curtis dissimilarity, weighted UniFrac distance, and unweighted UniFrac distance measures were used to calculate beta diversity. At *p* < 0.05, the differences were deemed significant. A Benjamini-Hochberg false discovery rate (FDR) correction was applied to the resultant *p*-value (24).

### 2.9 Statistical analysis

All data were analyzed using two-way ANOVA with interaction when the assumptions were satisfied. Tukey’s HSD test was used to investigate whether there were significant differences between treatment means. The Kruskal-Wallis test was used when ANOVA assumptions were violated. The relative abundance of bacterial communities was compared between groups across gut segments to determine whether Alterion would change the bacterial community structure. Alpha diversity indices were compared using two-way ANOVA, and multiple pairwise comparisons were conducted using the FDR method. R (Version 4.4.1) was used for all statistical analyses.

## 3 Results

### 3.1 Growth rate, feed intake, and feed conversion ratio

**Figure 1:**
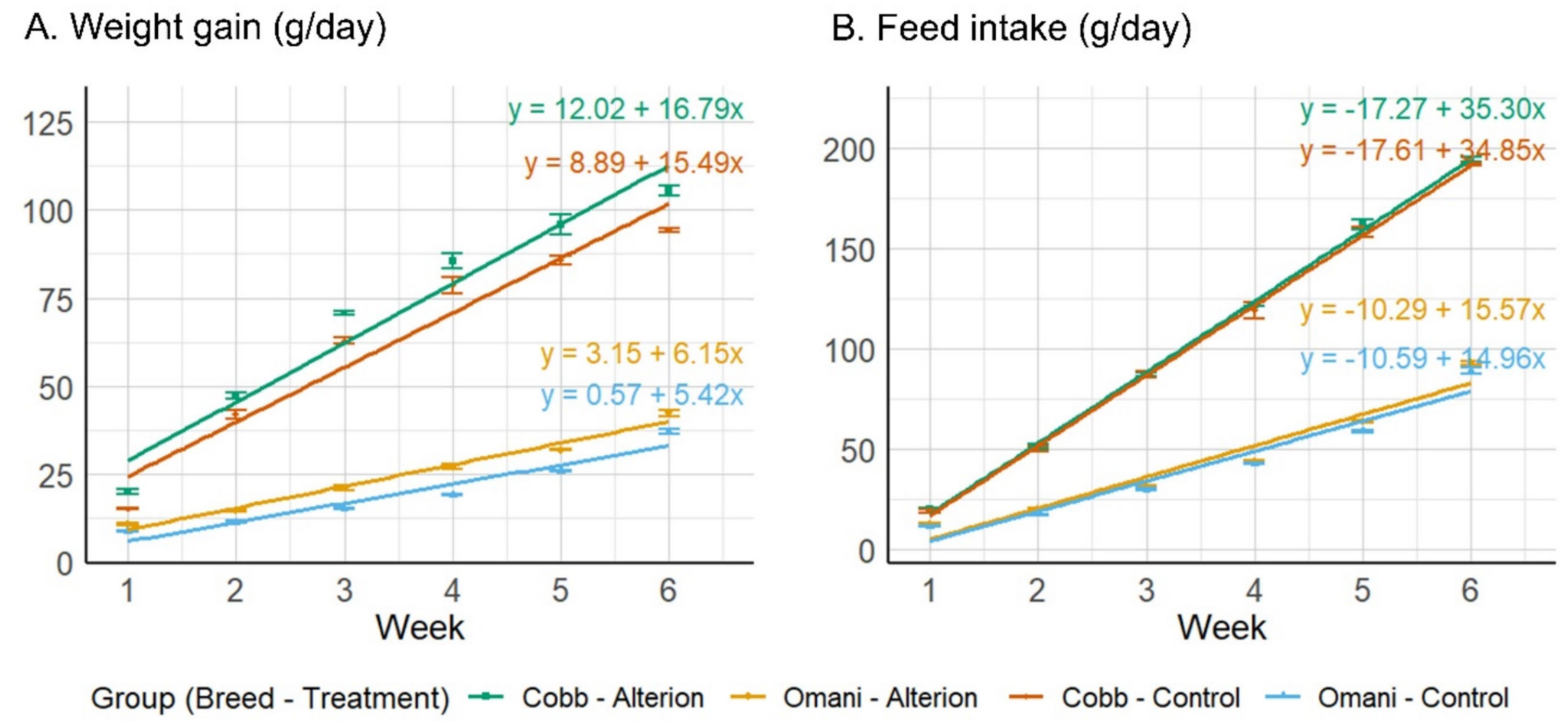
Mean weight gain **(A)** and feed intake **(B)** for chickens of the two breeds (Cobb 430 and Omani) under two treatments (Control and 0.05% Alterion) over the six-week rearing period. Each panel includes regression lines and equations (one for each breed-treatment combination) to describe the trends over time.

The results indicate that Alterion significantly enhanced body weight gain and FCR compared to the basal-diet groups (*p* < 0.001; Table 2). Specifically, broilers supplemented with Alterion demonstrated higher weight gains than the control group throughout weeks 1 to 6. Similarly, Omani chickens fed Alterion showed consistent weight gains during the same period. Specifically, the Alterion group showed a 12.1% increase in broilers and a 26.7% increase in Omani chickens. All differences were statistically significant (*p* < 0.001). Feed intake was significantly higher in Cobb 430 than in Omani chickens (*p* < 0.05), but there was no significant difference between the control and treatment groups of the same breed (*p* > 0.05; Table 2). The regression analysis of weight gain and feed intake revealed steeper slopes for Cobb 430 broilers than Omani chickens for both treatments (Control and 0.05% Alterion; Figure 1). The results indicated significant improvement in FCR in Cobb 430 broilers compared with Omani chickens in both the Alterion-supplemented and control groups (*p* < 0.001; Table 2).

**Table 2:** The impact of dietary Alterion on the feed intake (FI), daily gain (DG), and feed conversion ratio (FCR) of Cobb 430 and local chickens.

	Breed							
	Cobb 430		Omani					
	Alterion (g/kg)		Alterion (g/kg)			Significance		
Level	0.0	0.5	0.0	0.5	SEM	B	L	B*L
Week 1								
FI	19.25^a^	20.62^a^	11.86^b^	12.59^b^	0.525	***	NS	NS
DG	17.23^b^	20.02^a^	8.679^d^	10.81^c^	0.474	***	***	NS
FCR	1.11^bc^	1.04^c^	1.36^a^	1.16^b^	0.025	***	***	**
Week 2								
FI	50.21^a^	51.31^a^	17.61^b^	20.32^b^	0.819	***	*	NS
DG	42.07^b^	47.15^a^	11.36^d^	14.71^c^	0.792	***	***	NS
FCR	1.19^c^	1.08^d^	1.55^a^	1.38^b^	0.027	***	***	NS
Week 3								
FI	87.23^a^	87.33^a^	29.99^b^	31.57^b^	0.897	***	NS	NS
DG	62.89^b^	70.80^a^	15.08^d^	21.08^c^	0.717	***	***	NS
FCR	1.38^c^	1.23^d^	1.99^a^	1.52^b^	0.035	***	***	***
Week 4								
FI	119.21^a^	122.15^a^	42.99^b^	43.92^b^	2.034	***	NS	NS
DG	78.58^b^	85.50^a^	19.09^d^	27.14^c^	1.584	***	***	NS
FCR	1.52^bc^	1.42^c^	2.25^a^	1.62^b^	0.032	***	***	***
Week 5								
FI	158.31^a^	161.88^a^	59.03^b^	63.916^b^	1.797	***	*	NS
DG	85.72^b^	95.82^a^	25.92^d^	31.92^c^	1.514	***	***	NS
FCR	1.84^c^	1.69^d^	2.28^a^	2.00^b^	0.033	***	***	NS
Week 6								
FI	191.93^a^	194.41^a^	89.11^b^	92.96^b^	1.082	***	**	*
DG	94.28^b^	105.39^a^	37.05^d^	42.29^c^	0.907	***	***	**
FCR	2.03^c^	1.84^d^	2.40^a^	2.20^b^	0.041	***	***	NS
Overall								
FI	104.70^a^	106.28^a^	41.87^b^	44.21^b^	0.519	***	**	NS
DG	63.117^b^	70.776^a^	19.53^d^	24.66^c^	0.444	***	***	**
FCR	1.65^c^	1.50^d^	2.14^a^	1.795^b^	0.017	***	***	***

^a–d^Different letters within the same row show statistical significance (*p* < 0.05). Significance codes: *p* < 0.05, *; *p* < 0.01, **; *p* < 0.001, ***. NS, not significant. SEM, standard error of the means. B, breed; L, level; B*L, breed-level interaction.

### 3.2 Hematology and serum chemistry indices

**Figure 2:**
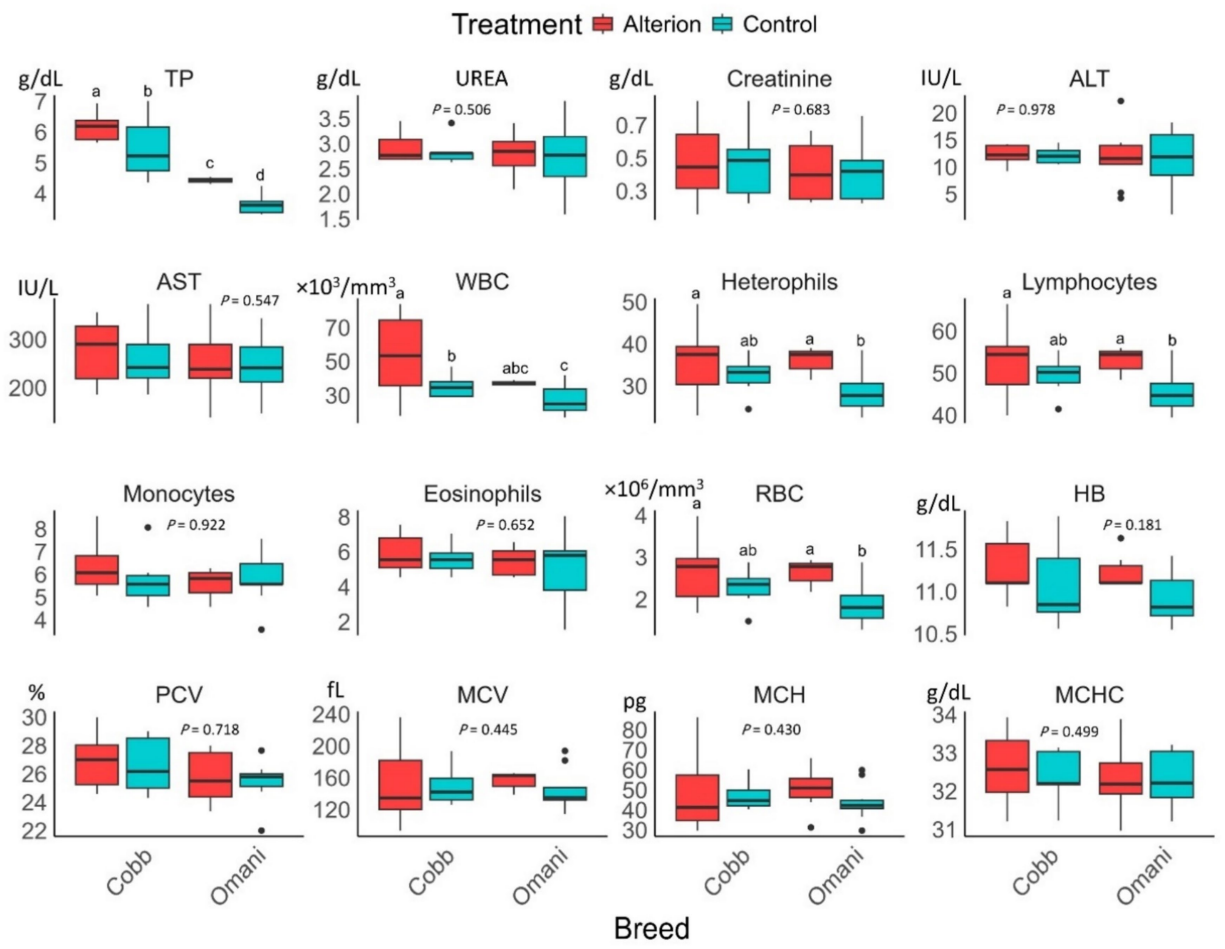
Effect of dietary Alterion on blood and serum chemistry indicators of Cobb 430 and local Omani chickens. Significance (*p* < 0.05) is indicated by different letters (a, b, c, d).

Alterion supplementation significantly increased TP and WBC levels in Cobb 430 and native Omani chickens (*p* < 0.05; Figure 2). RBCs, heterophils, and lymphocyte levels were significantly lower in control local chickens than in other groups (*p* < 0.05). However, the levels of urea, creatinine, ALT, AST, monocytes, eosinophils, hemoglobin, PCV, MCV, MCH, and MCHC were not affected by Alterion supplementation in both Cobb 430 broilers and local chickens (*p* > 0.05).

### 3.3 Intestinal morphometrics

**Figure 3:**
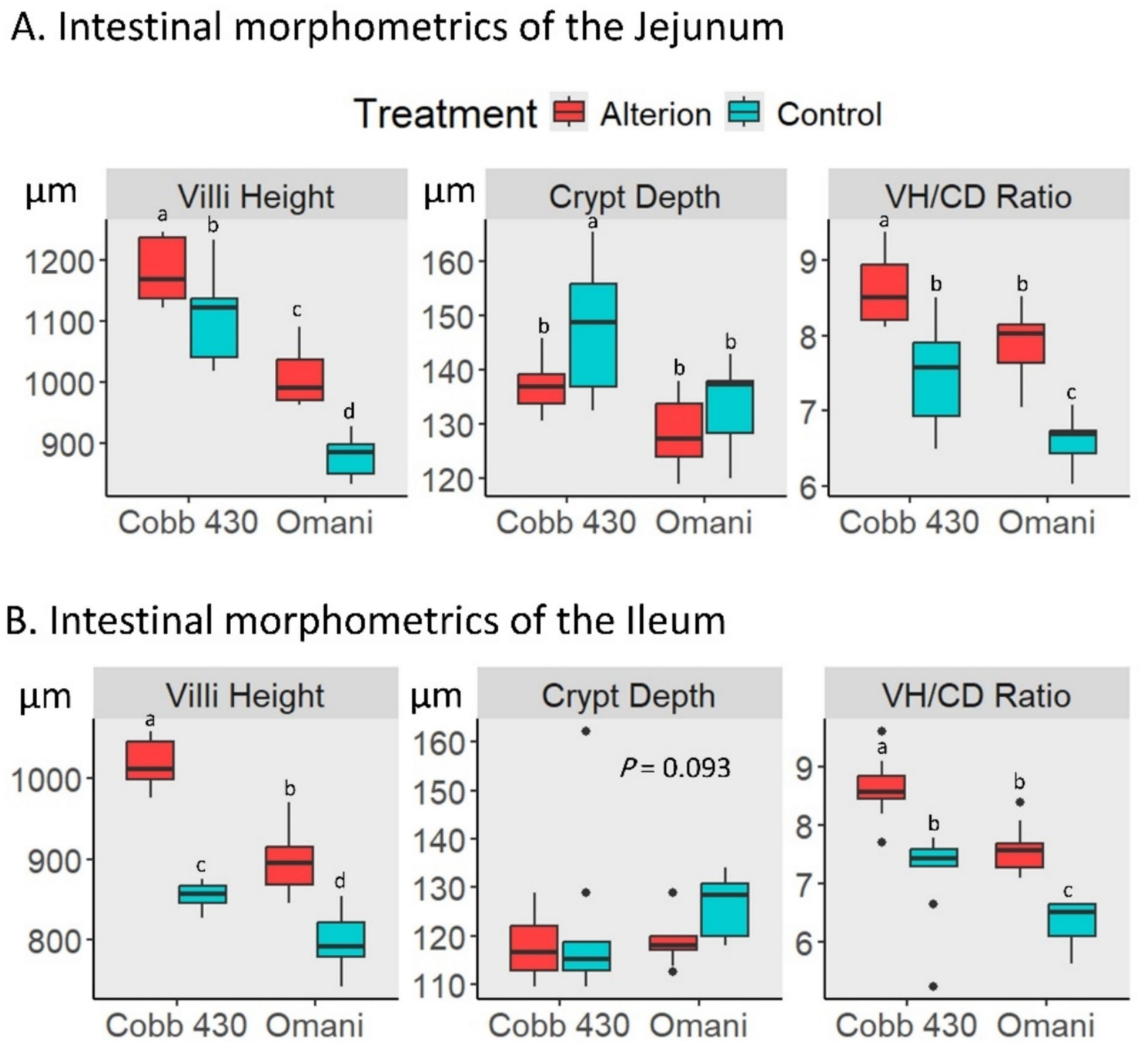
Effect of dietary Alterion on intestinal morphometrics in the jejunum **(A)** and ileum **(B)** of Cobb 430 and local Omani chickens. These metrics include villi height (VH), crypt depth (CD), and VH/CD ratio. Significance (*p* < 0.05) is indicated by different letters (a, b, c, d).

Cobb 430 control birds showed significantly higher VH and VH/CD ratios in the jejunum and ileum than Omani control chickens (*p* < 0.05; Figure 3). Furthermore, Alterion significantly increased VH and VH/CD ratio in both the jejunum and ileum of Cobb 430 and Omani chickens when compared with controls (*p* < 0.05). Conversely, Alterion supplementation significantly reduced the CD in the jejunum of Cobb 430 broilers (*p* < 0.05), whereas no significant difference was detected for CD in the ileum (*p* > 0.05).

### 3.4 Weight of carcass and internal organs

**Figure 4:**
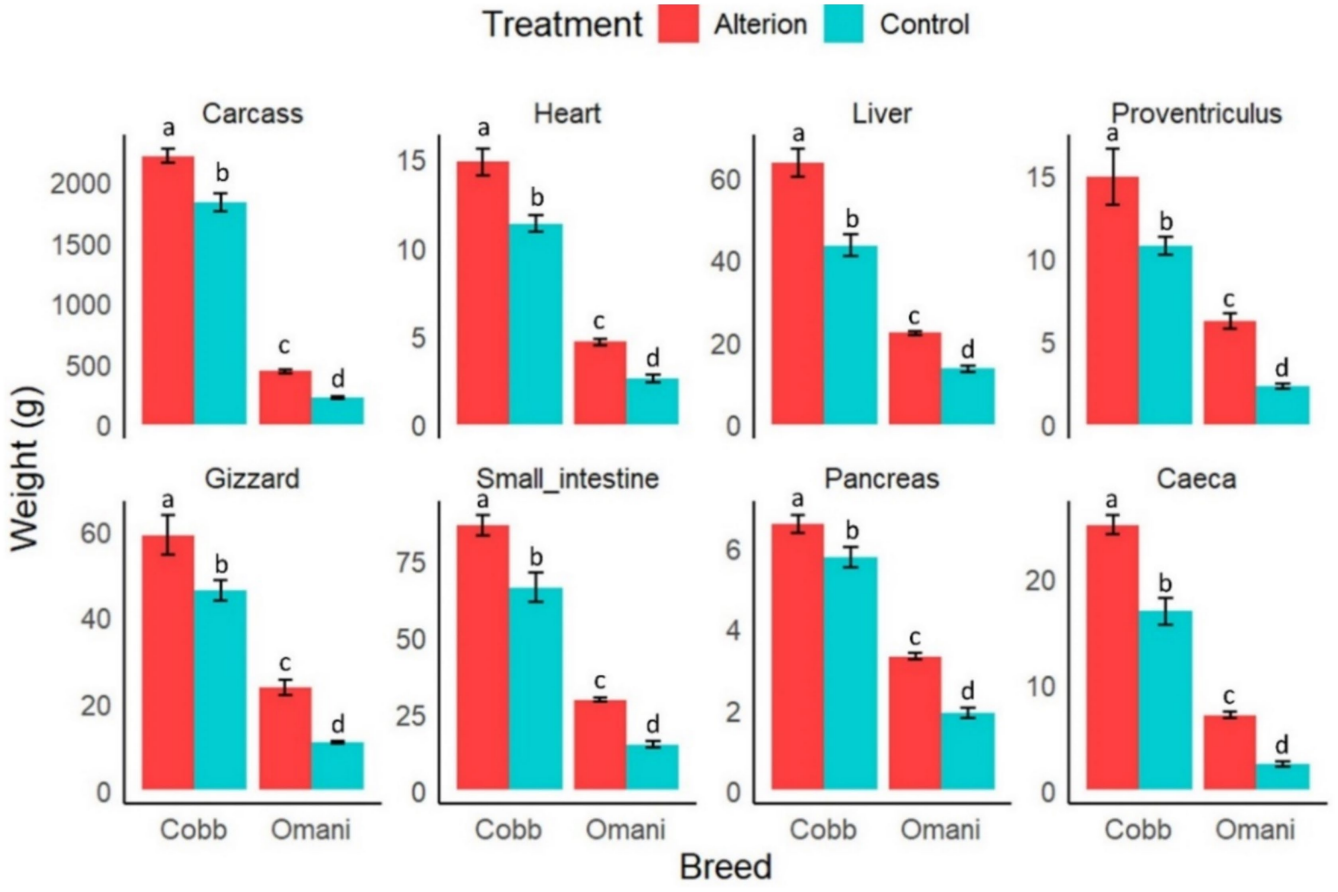
Effect of dietary Alterion on carcass yield and internal organ weights of Cobb 430 and local Omani chickens. Significance (*p* < 0.05) is indicated by different letters (a, b, c, d).

Both the chicken breed and dietary supplementation with Alterion significantly influenced carcass yield and internal organ weights (*p* < 0.05; Figure 4). Cobb 430 and local chickens fed the supplemented diets showed significantly higher internal organ weights and carcass yield (*p* < 0.05). Conversely, local chickens fed the basal diet showed a significantly reduced carcass yield and internal organ weights compared to the other groups (*p* < 0.05).

### 3.5 Meat quality characteristics

**Figure 5:**
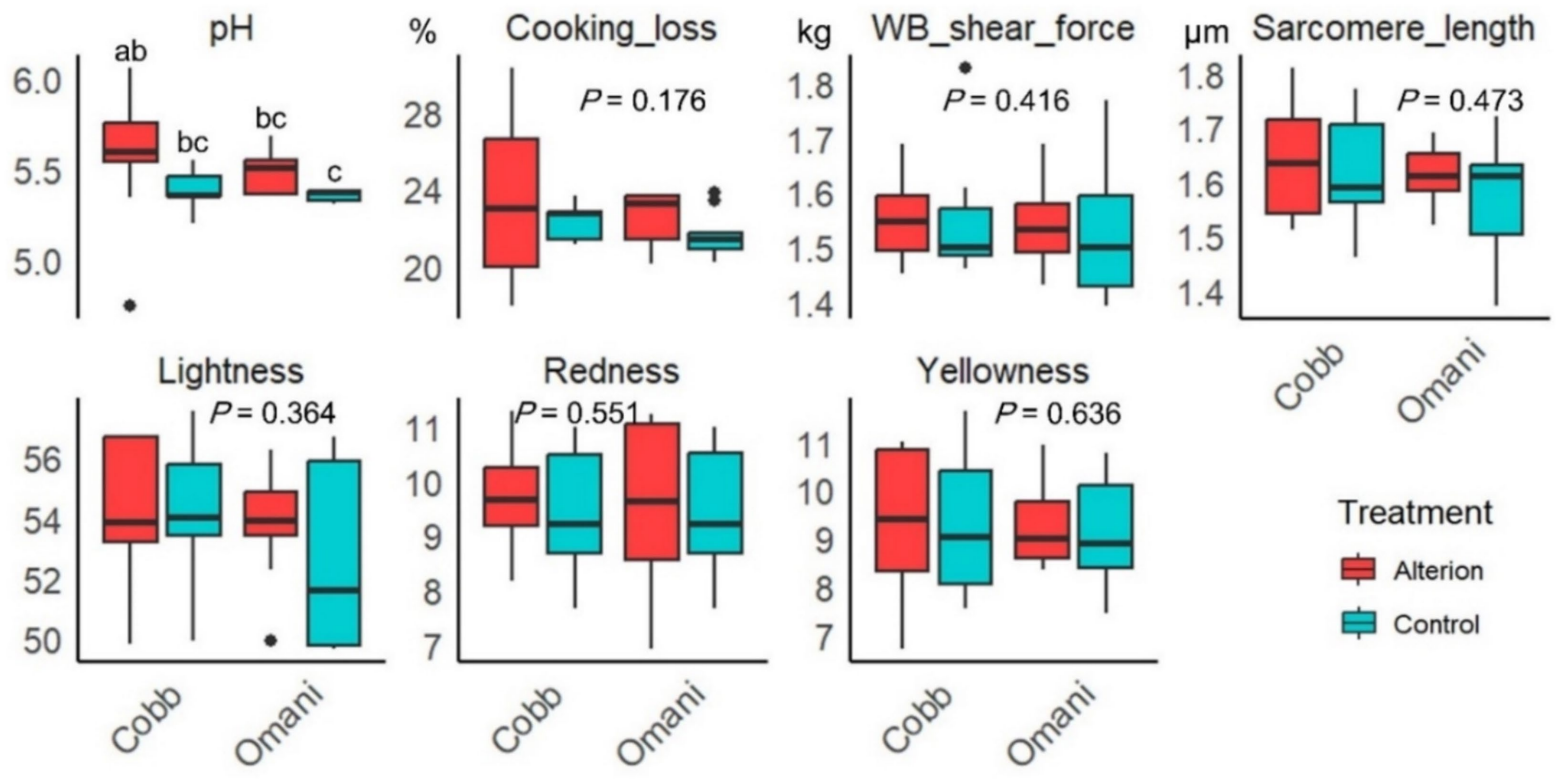
Effect of dietary Alterion on meat quality parameters of Cobb 430 and local Omani chickens. Significance (*p* < 0.05) is indicated by different letters (a, b, c, d).

Alterion showed no significant effect on all meat quality parameters except for pH, which was significantly elevated in Cobb 430 broilers supplemented with Alterion compared to the local control Omani chickens (*p* < 0.05; Figure 5).

### 3.6 Metagenomic study

#### 3.6.1 Sequence assembly and statistics

In Cobb 430 broiler chickens, a total of 3,788,897 sequences were obtained from 72 samples, with a mean and standard error of the mean (SEM) of 52,623.5 ± 479.7 sequences after quality filtering (Supplementary Table 1A). These sequences were grouped at a 97% sequence similarity level into a total of 21,306 OTUs, with a mean and SEM of 295.9 ± 10.4 OTUs per sample. In local Omani chickens, a total of 3,417,436 sequences were obtained from 72 samples, with a mean and SEM of 47,464.4 ± 347.1 sequences after quality filtering (Supplementary Table 1B). These sequences were grouped at a 97% sequence similarity level into a total of 24,992 OTUs, with a mean and SEM of 297.4 ± 14.0 OTUs per sample.

#### 3.6.2 Alpha diversity indices

**Figure 6:**
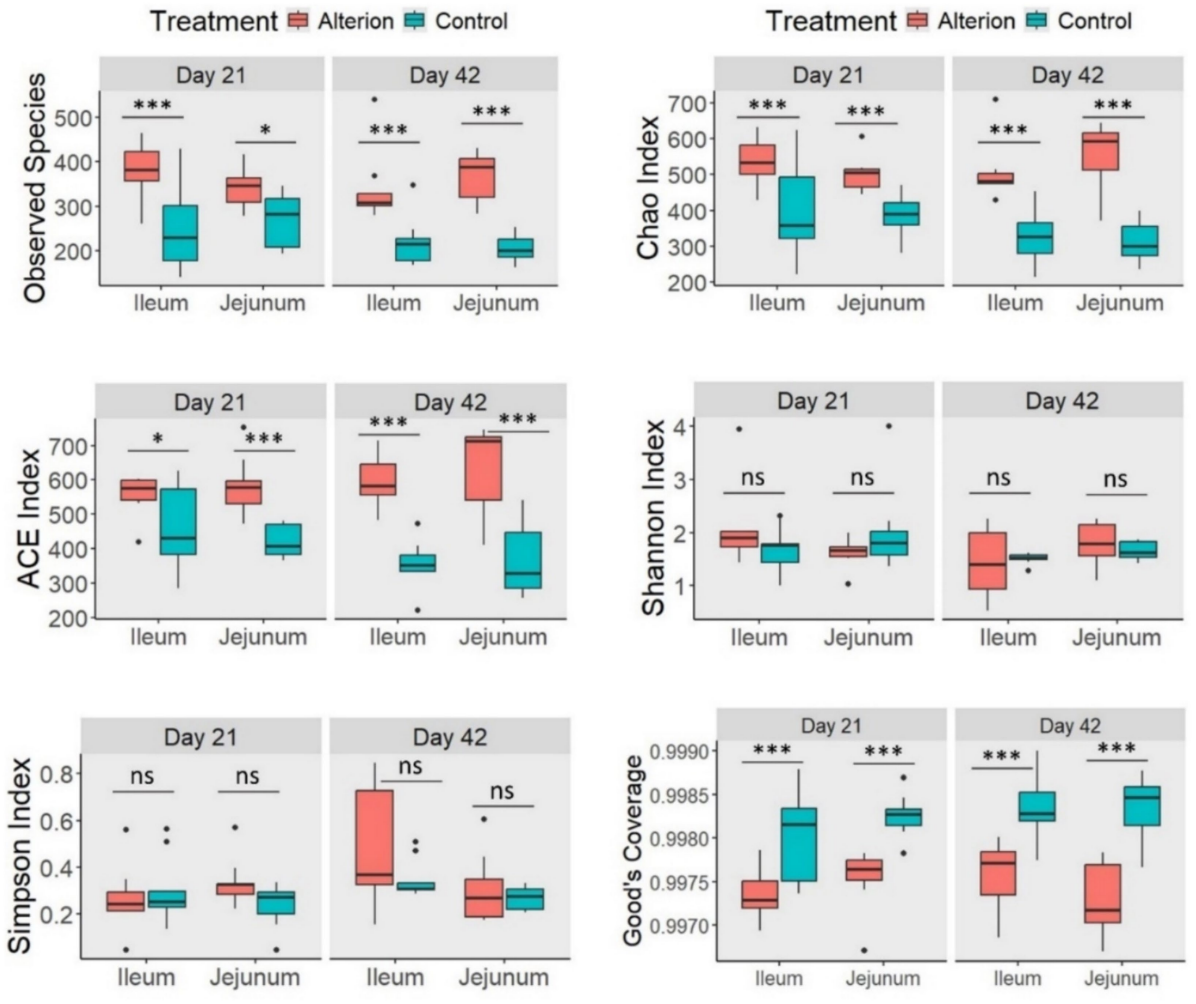
Alpha-diversity indices of gut microbiota in the jejunum and ileum of Cobb 430 broilers on days 21 and 42 following Alterion supplementation with 0.05% compared with control chickens that received only the basal diet. Significance levels include: *p* < 0.05 (*), *p* < 0.01 (**), *p* < 0.001 (***), and non-significant (ns).

Alterion supplementation significantly influenced several alpha diversity indices in Cobb 430 chickens (Figure 6). For example, the Observed Species index was remarkably higher (*p* < 0.05 to *p* < 0.001) in the Alterion group than in the control group at both sampling timepoints (days 21 and 42) and gut segments (ileum and jejunum). A similar trend was observed for the Chao index (*p* < 0.001) and ACE index (*p* < 0.05 to *p* < 0.001). Conversely, good’s coverage was significantly higher in the control group than in the Alterion treatment across all comparisons (*p* < 0.001). Neither Shannon nor Simpson indices were altered by Alterion treatment (*p* > 0.05).

**Figure 7:**
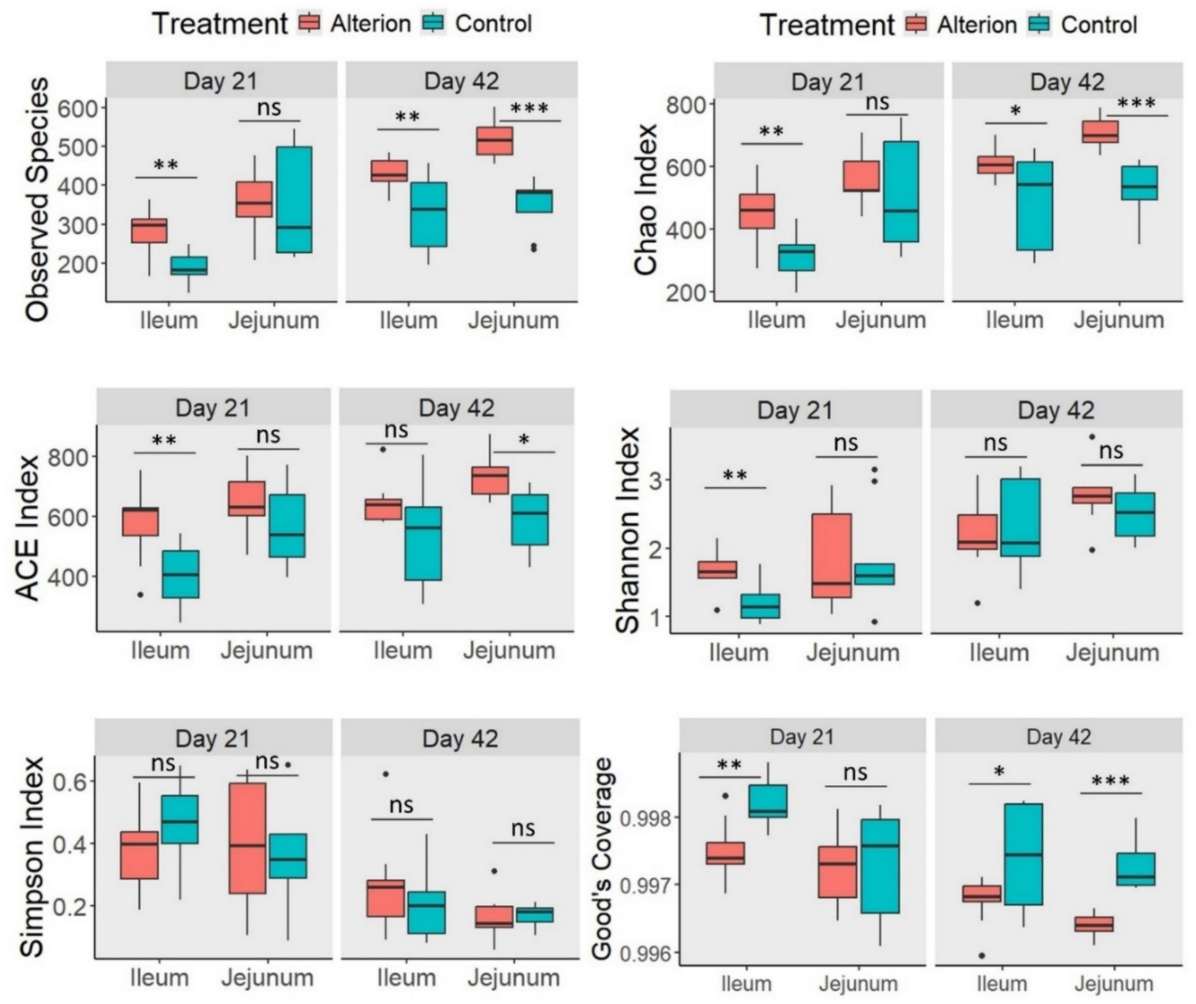
Alpha-diversity indices of gut microbiota in the jejunum and ileum of native Omani chickens on days 21 and 42 following Alterion supplementation (0.05%) compared with control chickens that received only the basal diet. Significance levels include: *p* < 0.05 (*), *p* < 0.01 (**), *p* < 0.001 (***), and non-significant (ns).

In Omani chickens, the observed species richness was significantly higher (*p* < 0.01) in the ileum on days 21 and 42 in the Alterion treatment compared to the control (Figure 7). In the jejunum, the observed species index was notably higher in the Alterion group on day 42 (*p* < 0.001), whereas no significant difference was observed on day 21 (*p* > 0.05). For the Chao index, all values were significantly higher in the Alterion group than in the controls (*p* < 0.05), except in the jejunum on day 21, where the difference was not significant (*p* > 0.05). The ACE index was significantly higher in the Alterion treatment than in the control group on day 21 in the ileum (*p* < 0.01) and on day 42 in the jejunum (*p* < 0.05). Furthermore, the Shannon index was significant (*p* < 0.01) only at the first sampling time point in the ileum. Conversely, no significant difference was detected for the Simpson index across treatments or gut segments (*p* > 0.05). Finally, Good’s coverage was significantly lower in the Alterion group in the ileum on day 21 (*p* < 0.01) and on day 42 in both the ileum and jejunum (*p* < 0.05 and *p* < 0.001, respectively).

#### 3.6.3 Analysis of similarity

The ANOSIM results revealed a moderate separation between groups in both the jejunum (*R* = 0.341, *p* = 0.001) and the ileum (*R* = 0.336, *p* = 0.001) of Cobb 430 chickens (Supplementary Figure S1A). Since Cobb 430 ANOSIM analysis exhibited moderate *R* values, we applied PERMANOVA analysis with 999 permutations for robustness, which also showed similar *R* and *p* values (Jejunum: *R* = 0.383, *p* = 0.001; Ileum: *R* = 0.357, *p* = 0.001). Conversely, Omani chickens exhibited a stronger separation between different groups in both the jejunum (*R* = 0.649, *p* = 0.001) and the ileum (*R* = 0.513, *p* = 0.001; Supplementary Figure S1B).

#### 3.6.4 Beta diversity indices

**Figure 8:**
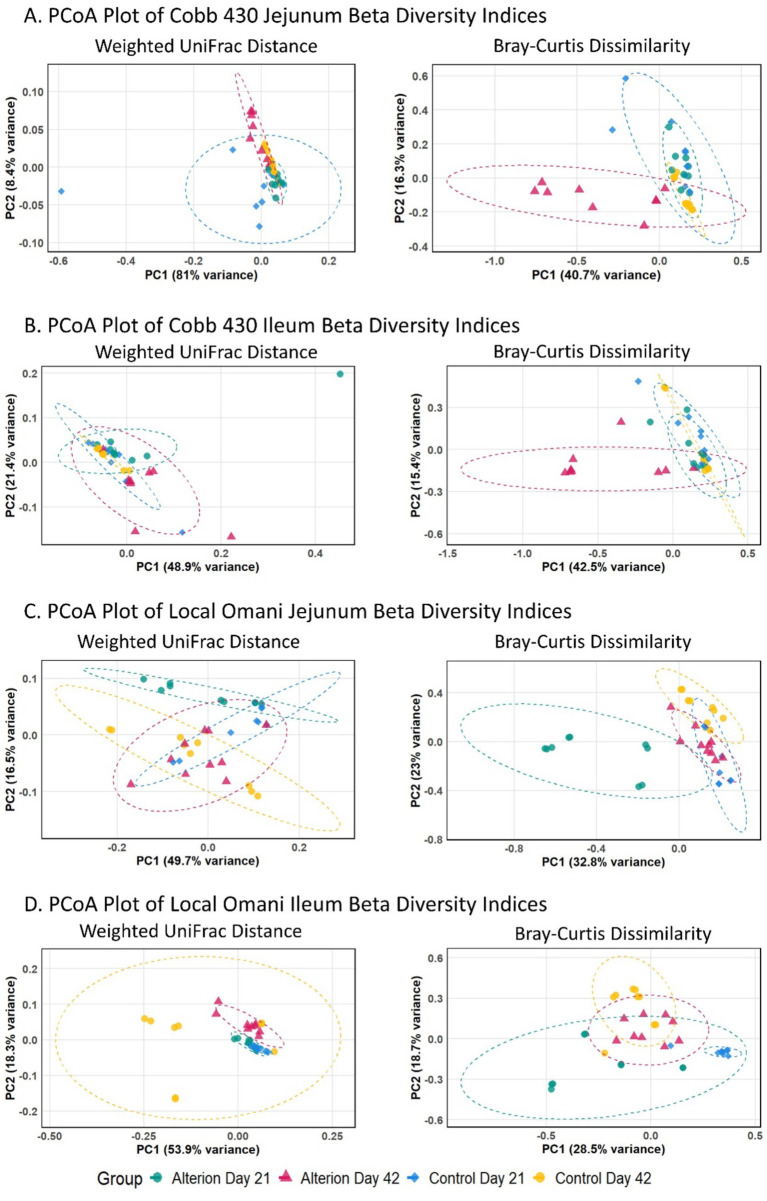
Principal coordinate analysis (PCoA) based on weighted UniFrac distance and Bray–Curtis dissimilarity of gut microbiota in Cobb 430 and local Omani chickens across the two dietary treatments (Control and 0.5% Alterion) for each intestinal segment (Jejunum and Ileum) at the two sampling time points (Days 21 and 42). Dashed ellipses represent the 95% confidence interval for each group, while the proportion of variance explained by PC1 and PC2 is indicated on the X and Y axes, respectively. **(A)** Cobb 430 jejunum, **(B)** Cobb 430 ileum, **(C)** Local Omani jejunum, **(D)** Local Omani ileum.

In Cobb 430 jejunum, Principal Coordinate Analysis (PCoA) revealed distinct clusters, with Alterion day 42 treatment and Control day 21 observed, PC1 and PC2 explaining most of the variance (Figure 8A), similar to Cobb 430 ileum (Figure 8B). These differences in clustering patterns were more obvious when the PCoA was calculated using unweighted UniFrac distance (Supplementary Figure S2A). In Omani chickens, different treatments clustered differently in the jejunum and ileum, with PC1 explaining about 50% of the weighted UniFrac distance and 30% of the Bray–Curtis dissimilarity (Figures 8C,D). Similarly, different treatments clustered distinctly for the unweighted UniFrac distance, but with lower proportions of variance explained by each PC (Supplementary Figure S2B). These results suggest shifts in gut microbiome composition due to bird age and Alterion supplementation.

#### 3.6.5 Distribution of OTUs among breeds, treatments, gut segments, and sampling time points

A comparison of the Cobb 430 control and Alterion groups at the two studied time points revealed a core bacterial microbiome of 564 shared OTUs, the highest number among all other comparisons (Supplementary Figure S3A). The control group exhibited the highest number of unique OTUs on day 42 (Supplementary Figure S3A). In the ileum, the core bacterial microbiome shared among all comparisons consisted of 226 OTUs, while the highest unique number (131 OTUs) occurred in the ileum of the control group on day 42 (Supplementary Figure S3B). Comparing gut segments across treatments with days combined revealed a core microbiome of 493 shared OTUs among all comparisons, with Alterion jejunum and Alterion ileum sharing 244 OTUs (Supplementary Figure S3C). In the jejunum, 213 OTUs were shared across treatments and days, compared with 59 and 93 unique OTUs in the Alterion treatment on days 21 and 42, respectively (Supplementary Figure S3D).

The results were similar in the local Omani chickens, although the number of OTUs was generally higher than in Cobb 430. For example, when considering treatments and gut segments together, the core bacterial microbiome shared between days 21 and 42 included 855 OTUs (Supplementary Figure S4A). In the ileum, 371 OTUs were shared among treatments across days, compared with 64 and 167 OTUs unique to the Alterion treatment on days 21 and 42, respectively (Supplementary Figure S4B). A comparison of the jejunum and ileum revealed 649 shared OTUs accompanied by 51 and 93 OTUs unique to the Alterion ileum and jejunum, respectively (Supplementary Figure S4C). Finally, the highest number of unique OTUs (210) was found in the jejunum segment of Alterion chickens on day 42, followed by 175 in the control jejunum on day 21 (Supplementary Figure S4D).

#### 3.6.6 Relative abundance of bacterial communities

##### 3.6.6.1 Class level

**Figure 9:**
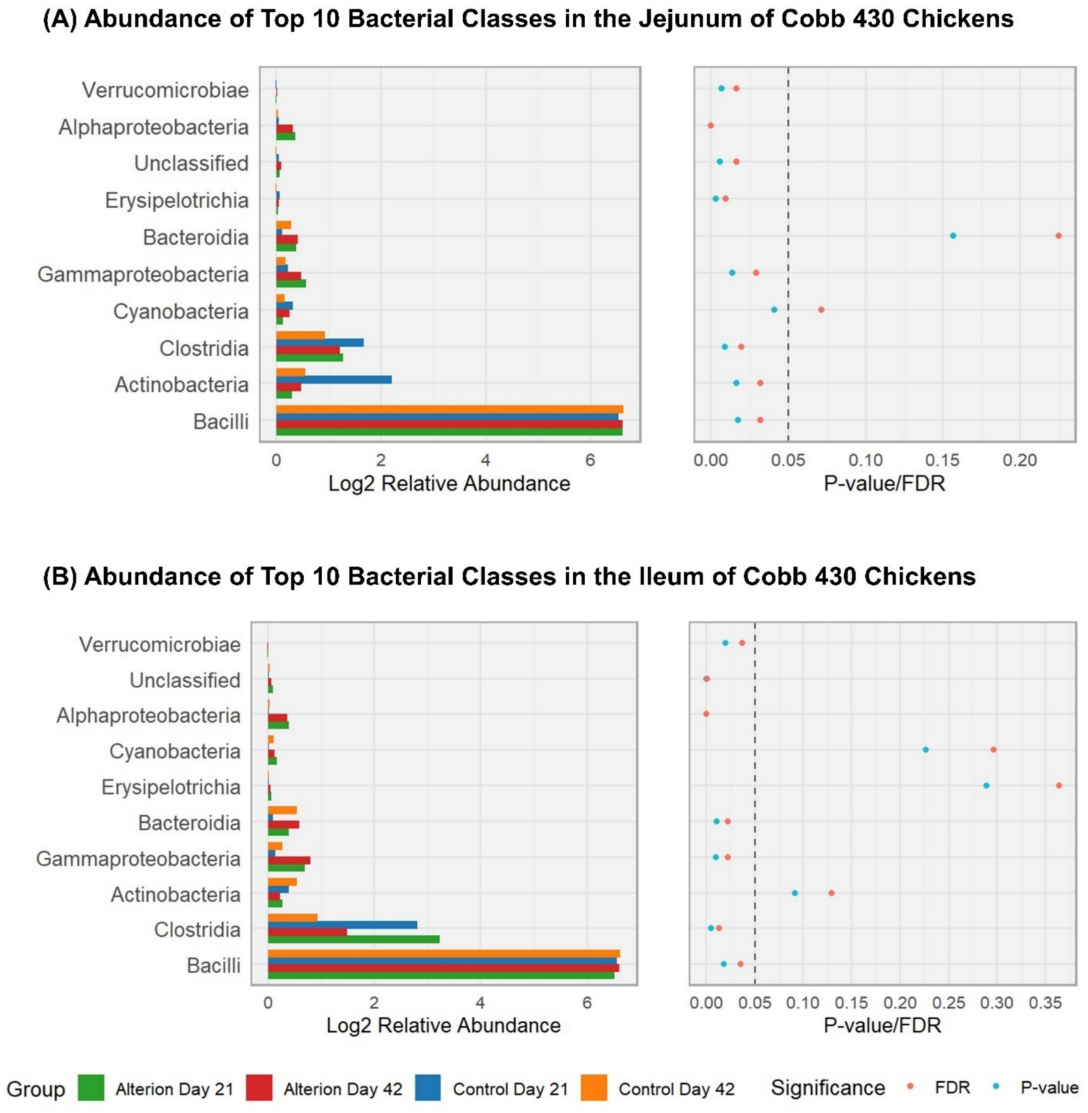
Relative abundance of the top 10 bacterial classes in the jejunum **(A)** and ileum **(B)** of Cobb 430 chickens under different treatments and time points (Control Day 21, Control Day 42, Alterion Day 21, and Alterion Day 42) and their statistical significance [*p*-value/False Discovery Rate (FDR)]. For optimum visualization, relative abundance data were log2-transformed before plotting. The dashed line represents the 0.05 *p*-value threshold.

In the jejunum of Cobb 430 chickens (Figure 9A), the three most abundant classes in all treatments were the Bacilli, Clostridia, and Actinobacteria. Bacilli were significantly lower (84.20%, *p* < 0.05) in the Control Day 21 group than the other three groups (97.93% for control day 42, 96.56% for Alterion day 21, and 96.43% for Alterion day 42). Conversely, Clostridia and Actinobacteria were significantly higher in the Control Day 21 group (9.17 and 5.25%, respectively; *p* < 0.05) than the other three groups. In the ileum of Cobb 430 chickens (Figure 9B), Bacilli were also the most abundant class (90.35% for Control Day 21; 97.11% for Control Day 42; 81.16% for Alterion Day 21; and 85.81% for Alterion Day 42; *p* < 0.05). Clostridia were the second most abundant class (5.82% for Control Day 21; 1.27% for Control Day 42; 16.21% for Alterion Day 21; and 6.41% for Alterion Day 42; *p* < 0.05). The relative abundance of Actinobacteria in the ileum of Cobb 430 broilers was not statistically significant among different treatments (*p* > 0.05; Figure 9B).

**Figure 10:**
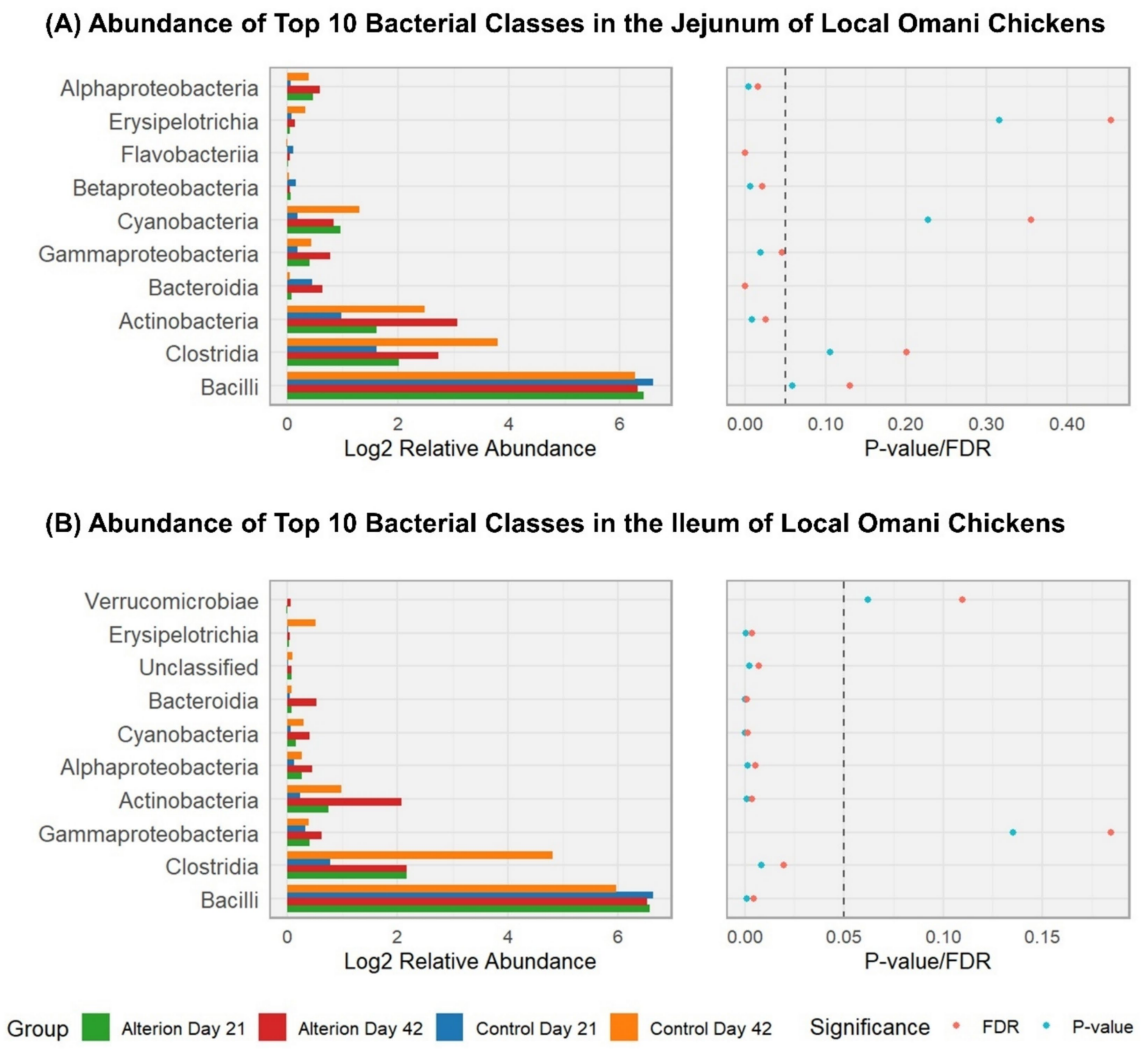
Relative abundance of the top 10 bacterial classes in the jejunum **(A)** and **(B)** ileum of local Omani chickens under different treatments and time points (Control Day 21, Control Day 42, Alterion Day 21, and Alterion Day 42) and their statistical significance [*p*-value/False Discovery Rate (FDR)]. For optimum visualization, relative abundance data were log2-transformed before plotting. The dashed line represents the 0.05 *p*-value threshold.

In the jejunum of Omani birds (Figure 10A), Bacilli were the most prevalent bacterial class (90.75% for Control Day 21; 75.99% for Control Day 42; 80.16% for Alterion Day 21; 77.01% for Alterion Day 42); however, these differences were not significant (*p* > 0.05). Clostridia were the second most abundant class, representing 3.85% in Control Day 21, 13.96% in Control Day 42, 8.63% in Alterion Day 21, and 9.41% in Alterion Day 42, but the differences were not significant (*p* > 0.05). The third most-abundant class was the Actinobacteria, representing 2.34% in Control Day 21, 7.05% in Control Day 42, 6.71% in Alterion Day 21, and 10.08% in Alterion Day 42 (*p* < 0.05). In the ileum of Omani chickens (Figure 10B), Bacilli were the most abundant class (97.26% for Control Day 21; 62.85% for Control Day 42; 93.35% for Alterion Day 21; 88.57% for Alterion Day 42; *p* < 0.001), followed by Clostridia (1.93% for Control Day 21; 21.33% for Control Day 42; 4.60% for Alterion Day 21; 4.32% for Alterion Day 42; *p* < 0.01). Actinobacteria were the third most abundant class in the ileum of Omani chickens (0.22% for Control Day 21; 6.79% for Control Day 42; 1.17% for Alterion Day 21; 4.60% for Alterion Day 42; *p* < 0.001).

##### 3.6.6.2 Genus level

The three most abundant bacterial genera in Cobb 430 jejunum included *Lactobacillus*, *Rothia*, and *Bifidobacterium* (Supplementary Figure S5A). *Lactobacillus* was the most abundant, representing 83.52% in Control Day 21, 97.27% in Control Day 42, 95.99% in Alterion Day 21, and 95.79% in Alterion Day 42; however, these differences were not statistically significant (*p* > 0.05). *Rothia* and *Bifidobacterium* were significantly higher in the Control Day 21 group (2.61 and 1.19%, respectively; *p* < 0.05) than in the other three Cobb 430 groups. In the ileum, *Lactobacillus* was the most prevalent genus in all treatments (Supplementary Figure S5B), representing 89.61% in Control Day 21, 96.45% in Control Day 42, 80.46% in Alterion Day 21, and 78.20% in Alterion Day 42 (*p* < 0.05).

In the jejunum of Omani chickens, *Lactobacillus* was the most abundant genus (Supplementary Figure S6A), accounting for 87.09% in Control Day 21, 63.71% in Control Day 42, 75.63% in Alterion Day 21, and 63.59% in Alterion Day 42 (*p* < 0.05). *Streptococcus* was the second most abundant genus on day 42 compared with day 21, regardless of Alterion supplementation (*p* < 0.001). Similarly, the relative abundance of *Bifidobacterium* was also higher on day 42 than on day 21 for both the control and Alterion, but the difference was not significant (*p* > 0.05). Comparable results were obtained for the ileum, where *Lactobacillus* was also the most abundant genus (96.56% for Control Day 21, 54.26% for Control Day 42, 86.45% for Alterion Day 21, 79.96% for Alterion Day 42; *p* < 0.001), followed by *Streptococcus* (0.36% for Control Day 21, 4.66% for Control Day 42, 0.66% for Alterion Day 21, 5.58% for Alterion Day 42; *p* < 0.001).

## 4 Discussion

Extensive research has explored the application of feed additives to enhance animal health and production outcomes (25–27). Nevertheless, investigations into their effects on slower-growing poultry breeds remain relatively scarce, as the majority of studies have concentrated on rapidly growing commercial lines (28). This study aimed to assess the influence of dietary supplementation with Alterion on the performance of Cobb 430 and Omani chickens raised under conventional Omani environmental conditions, characterized by ambient temperatures ranging between 23.5°C and 34.0°C. The results demonstrated that incorporating 0.05% Alterion into the diets of both breeds significantly enhanced growth performance and feed efficiency. The regression analysis reveals an improvement trend over time and can also be used to predict weight gain or feed intake values for each breed/treatment on any day from week 1 to week 6 using the corresponding equation.

These results agree with previous research on poultry and other livestock species, where dietary supplementation strategies, such as probiotics or protected amino acids, improved FCR, weight gain, and nitrogen utilization, largely due to improved nutrient digestibility and modulation of gut microbiota (29–31). Additional evidence suggests that the most substantial benefits occur when probiotics are administered from the first day of life (32). Sklan (33) emphasized that early access to feed supports post-hatch intestinal development. In the present study, breed-specific differences in feed intake emerged early, independent of the dietary treatment applied. This increased feed consumption was associated with the development of the digestive tract and elevated enzymatic activity related to digestion and metabolism (34). Blood profiling is widely recognized as a reliable method to evaluate birds’ physiological responses to environmental and dietary factors (35). Moreover, Muneer et al. (36) reported a direct association between diet quality and serum biochemical parameters. Supplementation with Alterion in this study modulated the hematological markers in both chicken breeds beneficially, with blood and serum values remaining within established reference ranges for broilers (37).

The observed rise in RBC counts following Alterion supplementation may contribute to enhanced oxygen transport, thereby supporting the birds’ overall physiological performance (37). Concurrent increases in hemoglobin concentrations further suggest improved oxygen delivery to body tissues, a critical factor in the optimal growth and development (37). Elevated WBC counts indicate immune system activation, which may increase resistance to pathogens and reduce the incidence of diseases. Such immunological improvements, alongside other physiological benefits, are likely linked to the antimicrobial and immunomodulatory effects of probiotics. These functional properties have been essential in minimizing the reliance on medically important antibiotic growth promoters in poultry production (38). Moreover, probiotic supplementation has been shown to positively influence immune regulation and promote intestinal health in chickens (39).

The increase in serum total protein levels in Alterion-supplemented birds can be attributed to the additive’s role in enhancing intestinal morphology, particularly through increased VH, which facilitates improved nutrient digestion and absorption within the small intestine. Enhanced absorption efficiency likely promotes greater uptake of amino acids and peptides, essential precursors for protein synthesis. Consequently, this improved nutrient assimilation may explain the higher circulating protein concentrations observed in the supplemented groups. These findings support the hypothesis that Alterion supplementation contributes to enhanced protein metabolism and biosynthesis in broiler and local Omani chickens. Similar outcomes have been reported in earlier studies, where feed additives improved protein utilization by optimizing digestive efficiency and nutrient availability (40, 41).

Furthermore, the present results demonstrate that Alterion supplementation has a positive influence on intestinal morphology in both chicken breeds. These improvements align with previous studies indicating that the inclusion of Alterion (0.01%) enhances FCR, promotes small intestinal development, and increases VH in broiler chickens (30, 42). Increased VH, reduced CD, along with a higher VH/CD ratio, indicate improved nutrient absorption efficiency, which is positively correlated with enhanced growth performance in chickens (43, 44). In particular, reduced CD is associated with a slower intestinal epithelial cell turnover, implying a decrease in metabolic demand (45, 46). This may account for the lower FCR observed in the treatment groups of both chicken breeds in the present study. Supporting this, prior investigations (47, 48) have reported that a diminished epithelial turnover rate reduces maintenance energy requirements, thereby facilitating more efficient growth. The observed improvements in intestinal histomorphology in broiler and Omani chickens, relative to controls, may be due to the beneficial effects of dietary additives on nutrient digestion and assimilation. Thus, these morphological enhancements likely contributed to improved feed utilization, resulting in increased weight gain and overall performance.

In addition, shorter villi and deeper crypts are typically correlated with a reduction in absorptive cells and an increase in secretory cells, which can affect nutrient absorption efficiency (49). Furthermore, changes in the structure of the intestinal mucosa may hinder nutrient absorption or increase the energy demands for intestinal maintenance (50). Previous studies have shown that deeper crypts stimulate crypt cell proliferation while reducing the synthesis and secretion of digestive enzymes, potentially impairing digestion (51). Enlarged crypts can lead to accelerated tissue turnover, raising nutrient requirements for tissue regeneration and thus decreasing nutrient absorption efficiency (52). The lower performance in the control groups of Cobb 430 and local birds may be due to the differences in the histological characteristics of the intestinal segments compared to those in the Alterion-supplemented groups. These findings align with those of Rysman et al. (44) and Ringenier et al. (53), who observed poor performance in broilers with shorter villi, deeper crypts, and a lower VH/CD ratio under field conditions.

A morphological study by Al-Marzooqi et al. (22) found that Cobb 500 chickens exhibited significantly greater VH than a local breed. This increase in VH is generally associated with enhanced digestive and absorptive functions, as it expands the surface area for absorption, boosts the expression of brush border enzymes, and improves nutrient transport mechanisms (54). The structure and enzymatic activity of enterocytes are critical aspects of intestinal mucosal function (55). Al-Marzooqi et al. (22) reported the significant impact of villus development on the growth performance of chickens.

The slower growth rate observed in local chickens in this study may be attributed, in part, to their lower feed intake. Even though feeder designs are optimized to minimize losses, local birds frequently behave like scavengers, resulting in feed waste. The growth of intestinal absorptive capacity is associated with changes in digestion and nutritional absorption (56). Young chicks’ villi grow more when feed is included in their diet, which increases their surface area and improves their capacity for absorption (57). To improve the growth performance of local chickens, it is recommended that a crossbreeding program be implemented that considers the intestinal developmental rates and the associated histological changes that influence intestinal function.

The current study also revealed significant differences in the bacterial microflora within each breed across various intestinal segments. These results suggest that each intestinal segment developed unique bacterial populations with distinct relative abundances (58). Although dietary supplementation had a minimal effect on the overall bacterial composition, it played a role in maintaining the normal ecological balance of the microbiota. These findings align with previous studies that have indicated the intestinal bacterial community is transient and evolves into a more stable population as the intestine develops (1, 2, 7).

Beta diversity analysis revealed the effect of Alterion on gut microbiome composition, with more pronounced effects in Cobb430 broilers, where Alterion showed a breed-specific or segment-specific impact on gut microbial community differing by age. Alpha-diversity indices, including observed species, Chao, and ACE, showed increased bacterial diversity in birds supplemented with Alterion at most timepoints, indicating that Alterion promoted the colonization of a broad range of microbial taxa. In addition, Shannon and Simpson’s indices indicated a more stable and even bacterial community, suggesting that Alterion increased the number of bacterial taxa without introducing severe shifts in the dominant species. Although Good’s coverage was significantly lower in the Alterion group than in control birds, it was still greater than 0.997 on average, suggesting richer microbial communities in the Alterion treatment. However, we suggest deeper sequencing coverage in future studies to ensure sufficient coverage of rare taxa. Overall, the results support the hypothesis that Alterion enhances microbial diversity and richness without disrupting microbial balance (30), which may explain the performance improvement reported in the current study.

## 5 Conclusion

The inclusion of Alterion as a feed additive has demonstrated several beneficial effects, including faster growth, better FCR, healthier intestinal morphology, and a positive impact on the structure and diversity of gut microbiota in Cobb 430 and native Omani chicken breeds. However, the impact of feed additives may vary depending on chicken breeds and environmental factors, such as housing conditions and climate. Therefore, results observed in one breed or environment may not be universally applicable. Factors like sample size, environmental settings, and the ability to generalize findings across various breeds should be considered. Future research should explore how these supplements influence intestinal mucosal responses and their potential role in protecting chickens from enteric infections. Furthermore, additional research is recommended to understand the mechanisms through which these supplements modulate the immune system in poultry.

## Data Availability

The raw sequencing data have been deposited at the NCBI Sequence Read Archive (SRA) database (BioProject: PRJNA1265125).
