# Supplementary material for: Beneficial effects of Alterion supplementation on growth metrics, intestinal histomorphology, and microbial communities in indigenous and commercial chicken breeds
Source: Front Vet Sci. 2025 Jul 11;12:1630712. doi: 10.3389/fvets.2025.1630712 (PMC12290415; doi:10.3389/fvets.2025.1630712)
Supplement: Supplementary file 2 [file Data_Sheet_1.docx]

**Supplementary Figures**


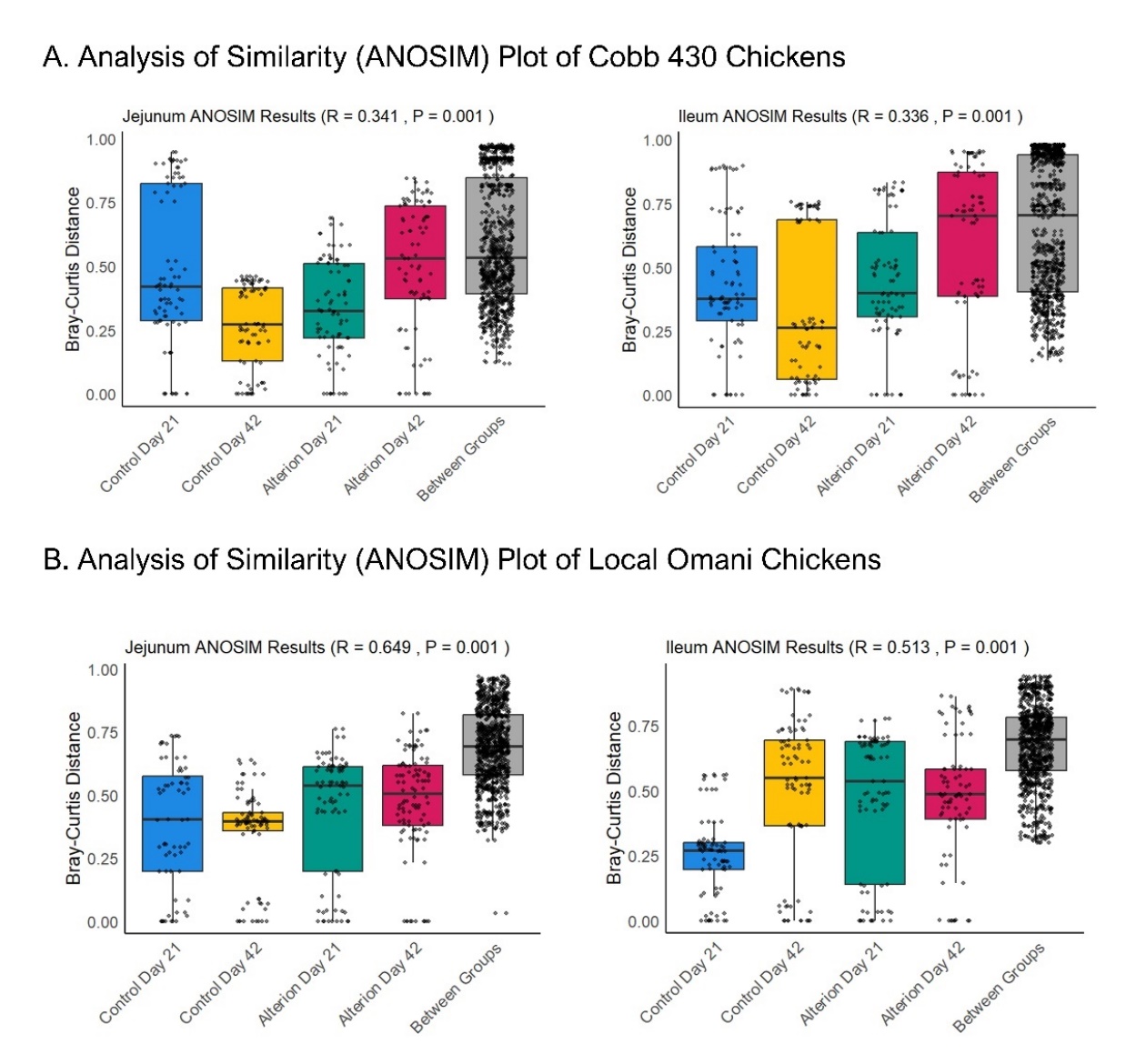


**Figure S1**. Analysis of Similarity (ANOSIM) plots based on Bray-Curtis dissimilarity of microbial communities in the jejunum (left panels) and ileum (right panels) of Cobb 430 and local Omani chickens supplemented with 0.05% Alterion compared with controls (basal diet). The higher the ANOSIM *R*-values, the stronger the microbial separation between treatment groups.


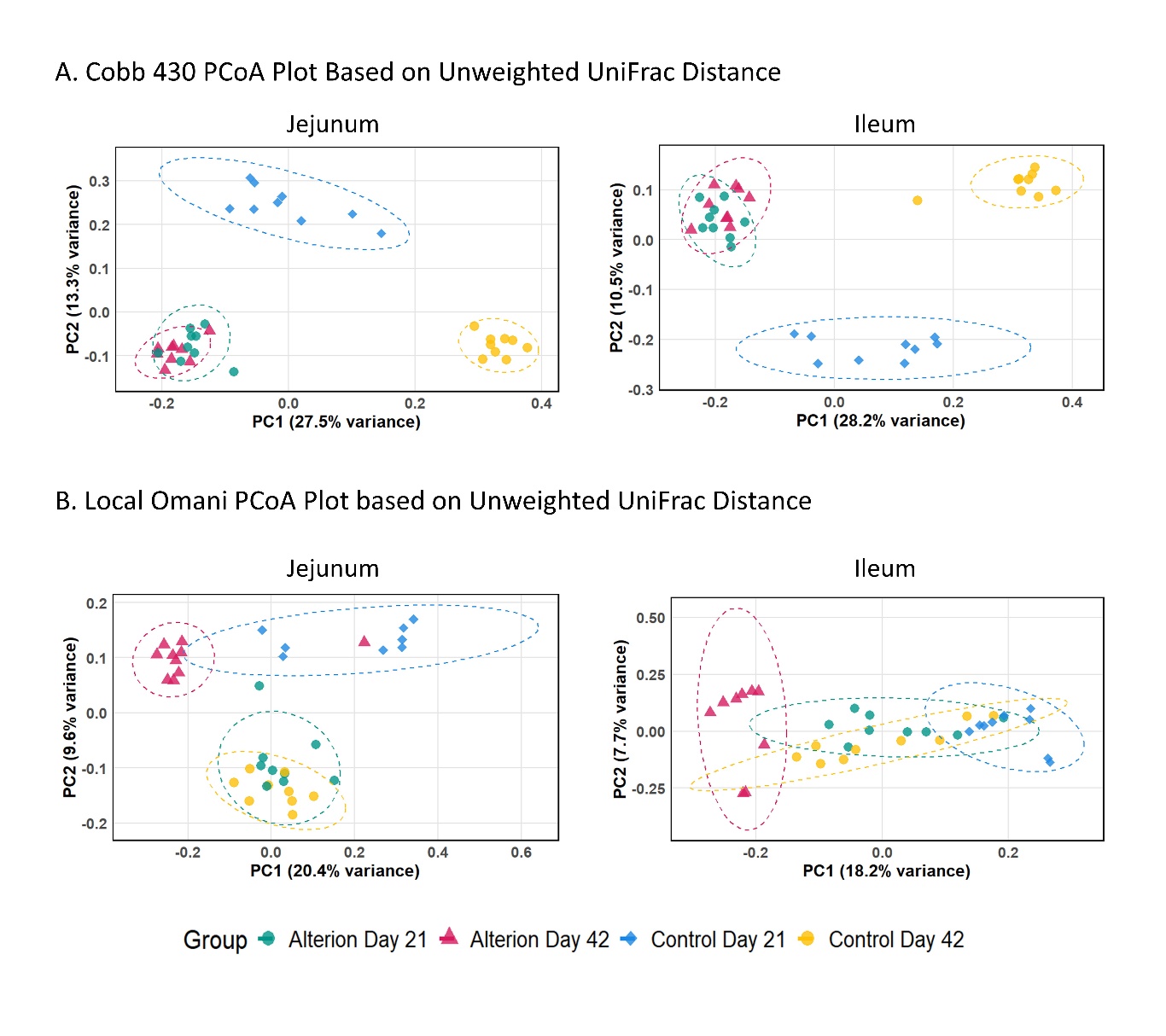


**Figure S2.** Principal Coordinate Analysis (PCoA) based on unweighted UniFrac distance of gut microbiota in Cobb 430 and local Omani chickens across the two dietary treatments (Control and 0.5% Alterion) for each intestinal segment (Jejunum and Ileum) at the two sampling time points (Days 21 and 42). Dashed ellipses represent the 95% confidence interval for each group, while the proportion of variance explained by PC1 and PC2 is indicated on the X and Y axes, respectively.


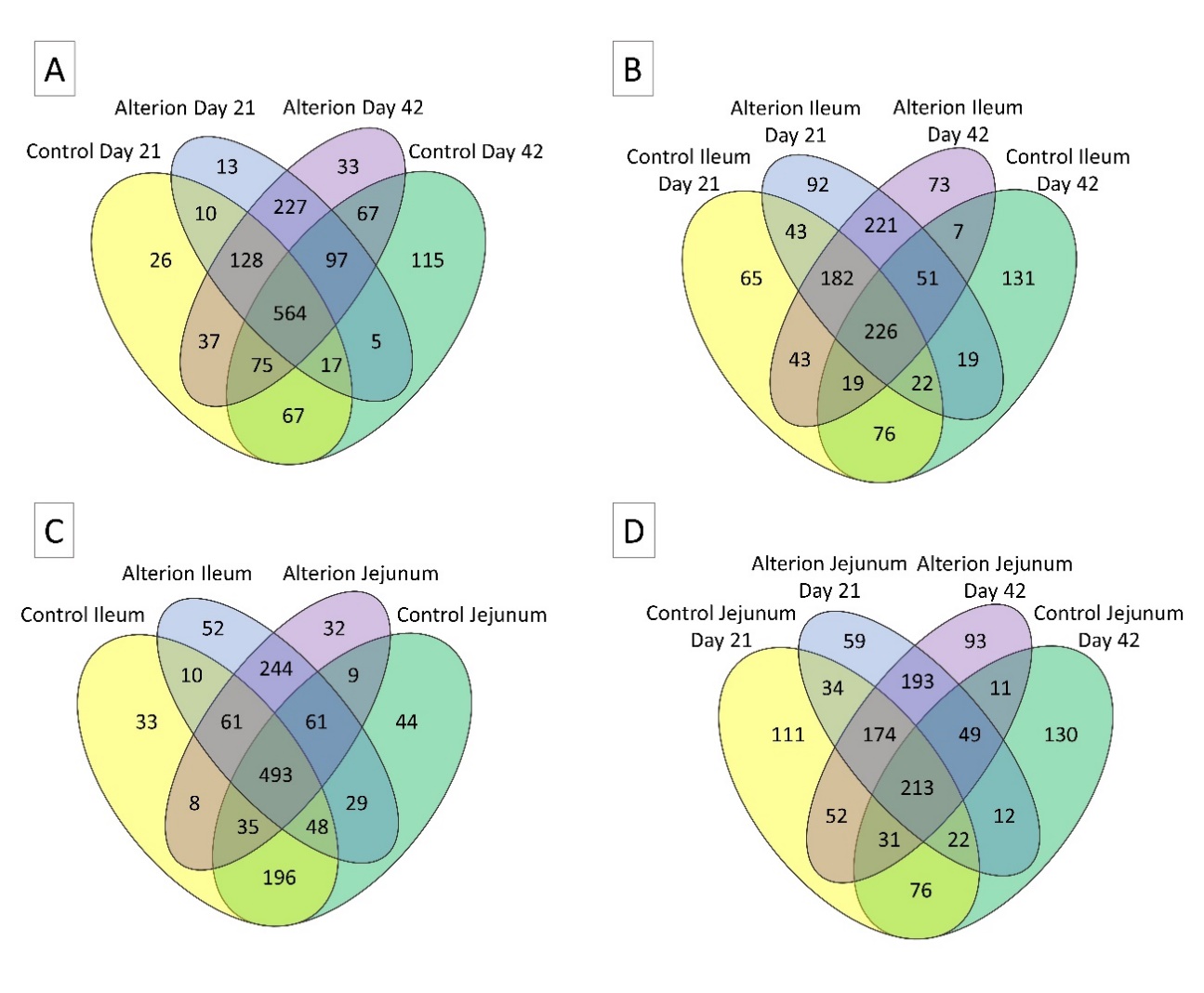


**Figure S3.** Venn diagram of shared and unique OTUs from the Cobb 430 chicken gut microbiome across the two dietary treatments (Control and 0.5% Alterion) for each intestinal segment (jejunum and ileum) at the two sampling time points. (A) Control and Alterion groups across days. (B) Control and Alterion groups in the ileum across days. (C) Control and Alterion groups in the ileum. (D) Control and Alterion groups in the jejunum across days.


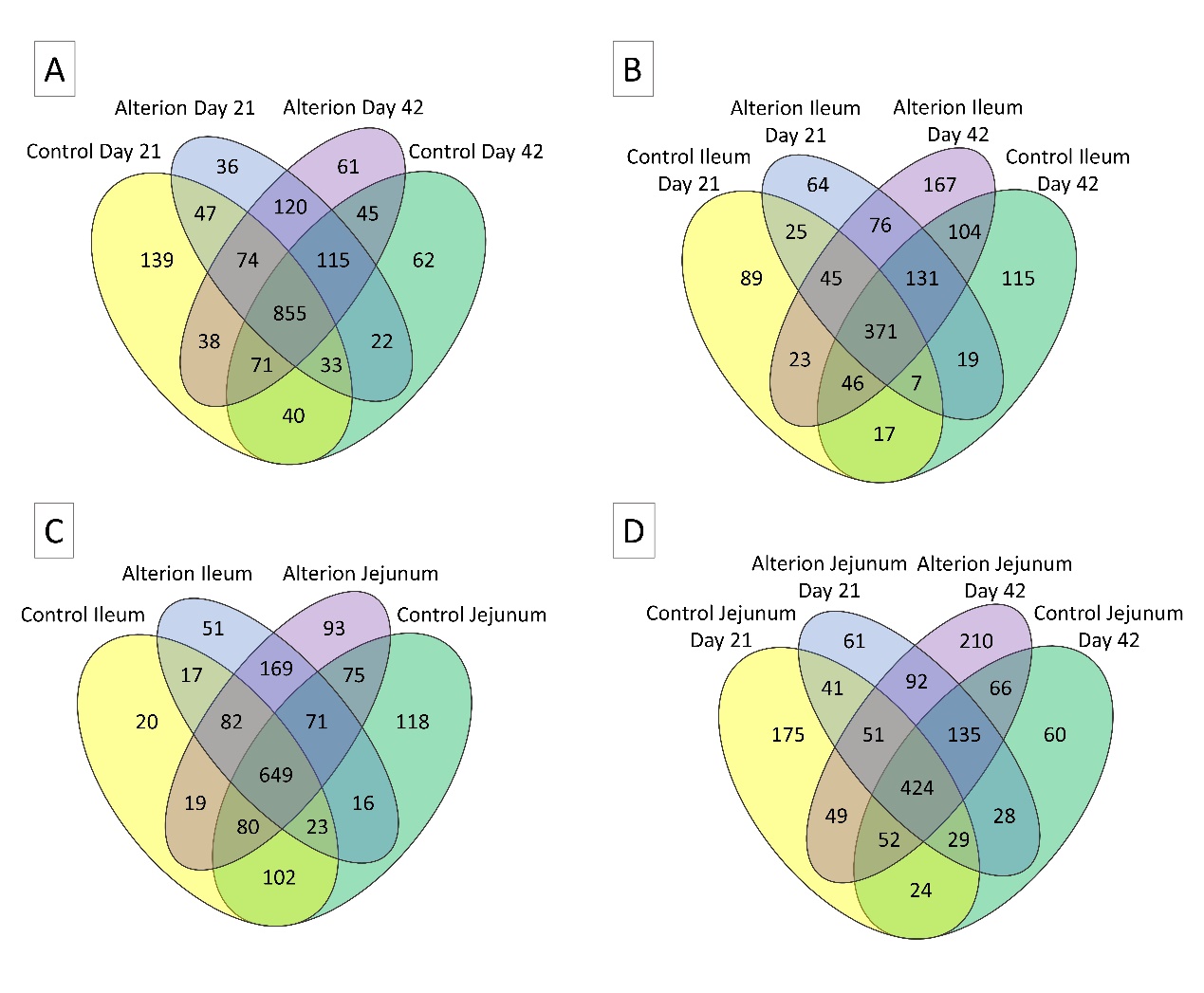


**Figure S4.** Venn diagram of shared and unique OTUs from the local Omani chicken gut microbiome across the two dietary treatments (Control and 0.5% Alterion) for each intestinal segment (jejunum and ileum) at the two sampling time points. (A) Control and Alterion groups across days. (B) Control and Alterion groups in the ileum across days. (C) Control and Alterion groups in the ileum. (D) Control and Alterion groups in the jejunum across days.


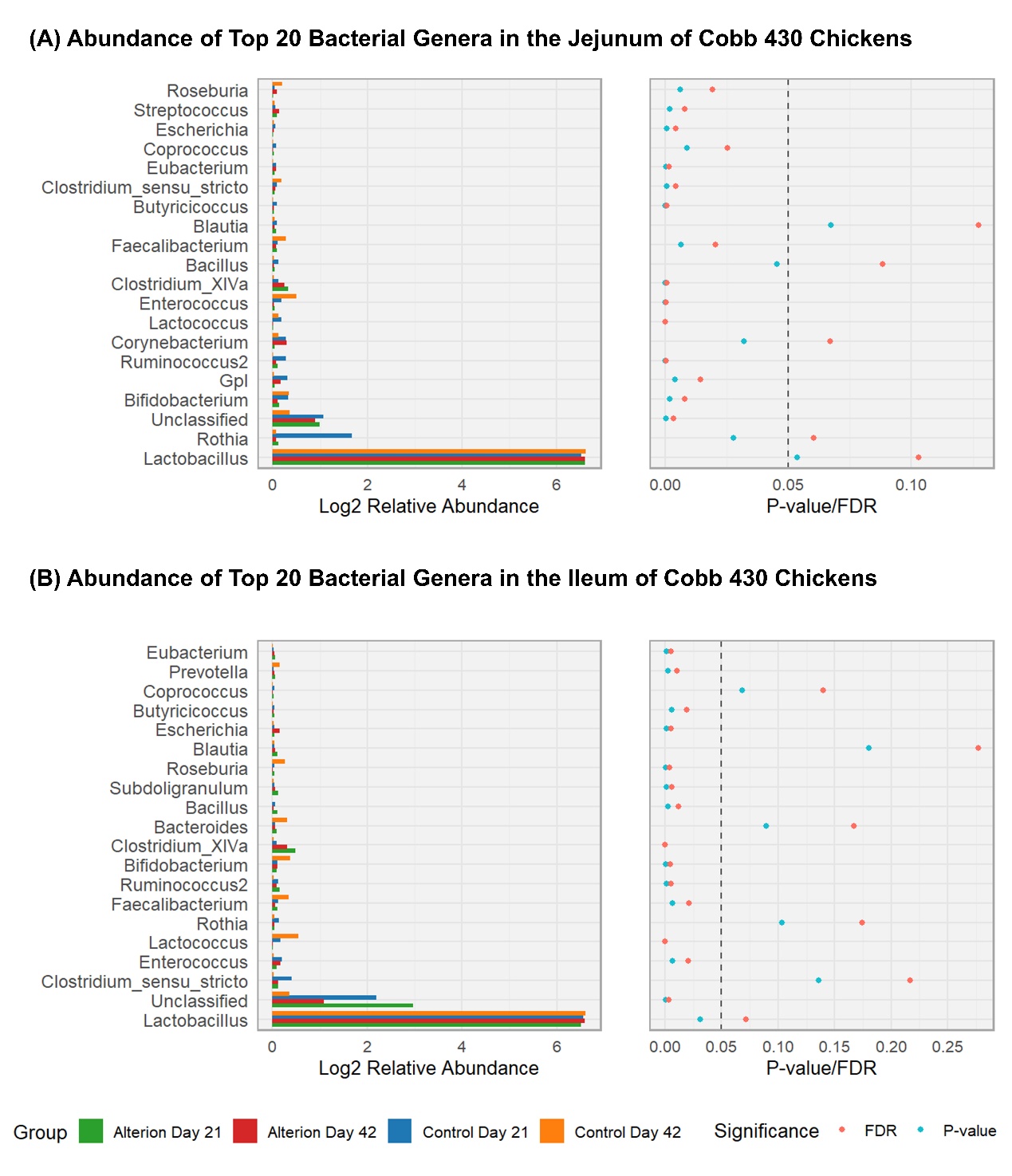


**Figure S5.** Relative abundance of the top 20 bacterial genera in the jejunum and ileum of Cobb 430 chickens under different treatments and time points (Control Day 21, Control Day 42, Alterion Day 21, and Alterion Day 42) and their statistical significance (*P*-value/False Discovery Rate (FDR)). For optimum visualization, relative abundance data were log2-transformed before plotting. The dashed line represents the 0.05 *P*-value threshold.


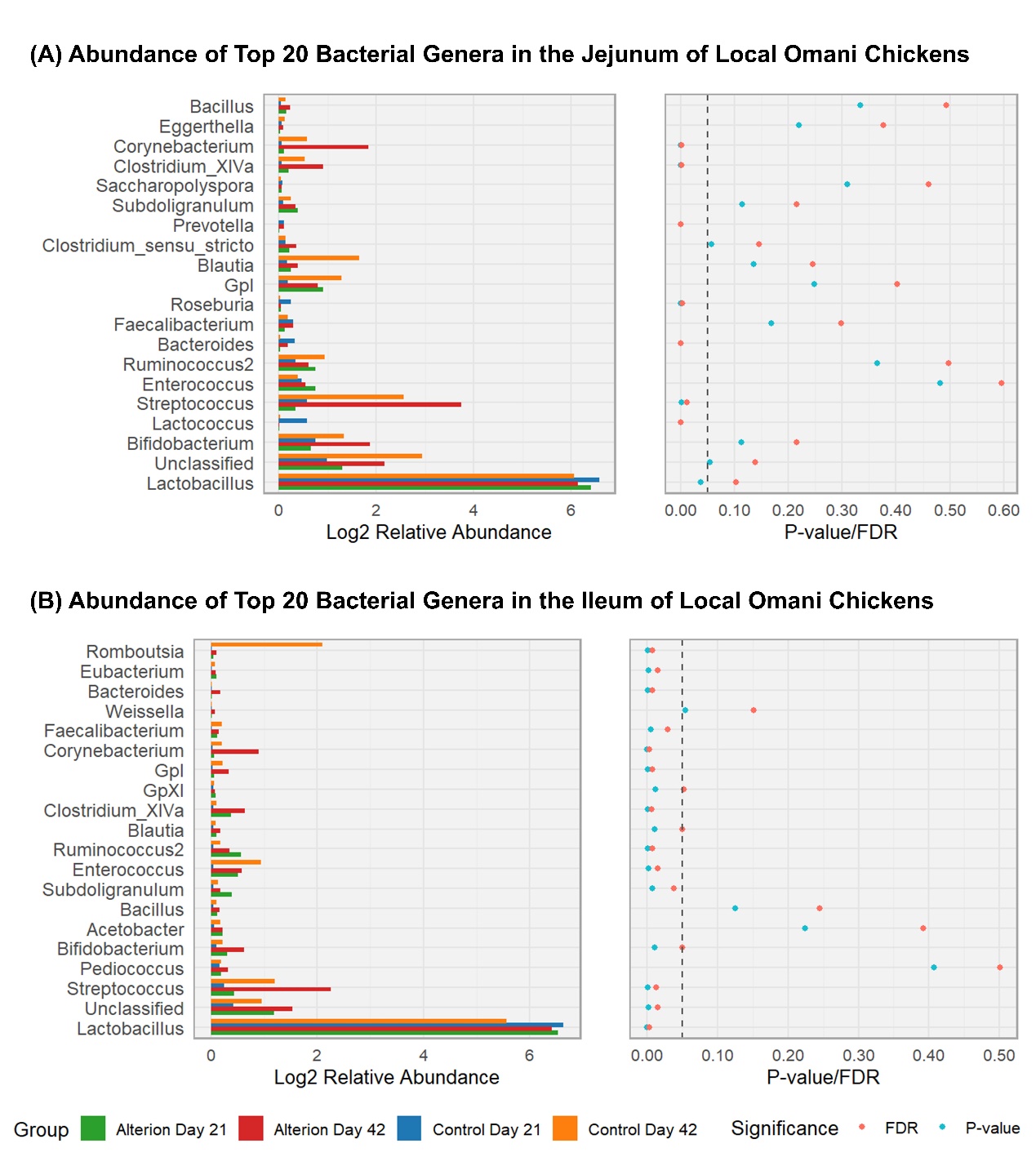


**Figure S6.** Relative abundance of the top 20 bacterial genera in the jejunum and ileum of local Omani chickens under different treatments and time points (Control Day 21, Control Day 42, Alterion Day 21, and Alterion Day 42) and their statistical significance (*P*-value/False Discovery Rate (FDR)). For optimum visualization, relative abundance data were log2-transformed before plotting. The dashed line represents the 0.05 *P*-value threshold.
